# High-speed tensor tomography: iterative reconstruction tensor tomography (IRTT) algorithm

**DOI:** 10.1107/S2053273318017394

**Published:** 2019-02-06

**Authors:** Zirui Gao, Manuel Guizar-Sicairos, Viviane Lutz-Bueno, Aileen Schröter, Marianne Liebi, Markus Rudin, Marios Georgiadis

**Affiliations:** a Paul Scherrer Institut, Villigen PSI, 5232, Switzerland; b Institute for Biomedical Engineering, ETH Zurich, Zurich, 8093, Switzerland; c Chalmers University of Technology, Gothenburg, SE-412 96, Sweden; d New York University Medical Center, New York, NY 10016, USA

**Keywords:** small-angle X-ray scattering, tensor tomography, iterative reconstruction algorithm

## Abstract

The recent advent of tensor tomography techniques has enabled tomographic investigations of the 3D nanostructure organization of biological and material science samples. These techniques extended the concept of conventional X-ray tomography by reconstructing not only a scalar value such as the attenuation coefficient per voxel, but also a set of parameters that capture the local anisotropy of nanostructures within every voxel of the sample. Tensor tomography data sets are intrinsically large as each pixel of a conventional X-ray projection is substituted by a scattering pattern, and projections have to be recorded at different sample angular orientations with several tilts of the rotation axis with respect to the X-ray propagation direction. Currently available reconstruction approaches for such large data sets are computationally expensive. Here, a novel, fast reconstruction algorithm, named iterative reconstruction tensor tomography (IRTT), is presented to simplify and accelerate tensor tomography reconstructions. IRTT is based on a second-rank tensor model to describe the anisotropy of the nanostructure in every voxel and on an iterative error backpropagation reconstruction algorithm to achieve high convergence speed. The feasibility and accuracy of IRTT are demonstrated by reconstructing the nanostructure anisotropy of three samples: a carbon fiber knot, a human bone trabecula specimen and a fixed mouse brain. Results and reconstruction speed were compared with those obtained by the small-angle scattering tensor tomography (SASTT) reconstruction method introduced by Liebi *et al.* [*Nature* (2015 ▸), **527**, 349–352]. The principal orientation of the nanostructure within each voxel revealed a high level of agreement between the two methods. Yet, for identical data sets and computer hardware used, IRTT was shown to be more than an order of magnitude faster. IRTT was found to yield robust results, it does not require prior knowledge of the sample for initializing parameters, and can be used in cases where simple anisotropy metrics are sufficient, *i.e.* the tensor approximation adequately captures the level of anisotropy and the dominant orientation within a voxel. In addition, by greatly accelerating the reconstruction, IRTT is particularly suitable for handling large tomographic data sets of samples with internal structure or as a real-time analysis tool during the experiment for online feedback during data acquisition. Alternatively, the IRTT results might be used as an initial guess for models capturing a higher complexity of structural anisotropy such as spherical harmonics based SASTT in Liebi *et al.* (2015 ▸), improving both overall convergence speed and robustness of the reconstruction.

## Abstract 2

A fast and robust reconstruction algorithm for small-angle scattering tensor tomography, named iterative reconstruction tensor tomography, is presented. It employs a second-rank tensor model and an iterative error backpropagation to simplify and accelerate tensor tomography reconstruction.

## 1. Introduction   

Information about micro- or nanoscopic structural organization within a macroscopic sample is often of great importance. For example, in material science, alignment of carbon nanotubes strongly influences the resistivity of nanotube films (Behnam *et al.*, 2007 ▸; Shekhar *et al.*, 2011 ▸) and molecular anisotropy in an additive manufacturing process is shown to be crucial to controlling structure and morphology (Ivanova *et al.*, 2013 ▸). In biology, structure is often optimized for its function (Fratzl & Weinkamer, 2007 ▸), and significant nano­structural alignment is found in many biological materials and tissues (Fratzl, 2012 ▸; Lichtenegger *et al.*, 1999 ▸; Meek & Boote, 2009 ▸; Masic *et al.*, 2015 ▸; Deymier-Black *et al.*, 2014 ▸). For instance, the orientation of mineralized collagen fibers in bone tissue determines its local mechanical properties (Martin & Ishida, 1989 ▸; Granke *et al.*, 2013 ▸), and is abnormal in different bone pathologies (Giannini *et al.*, 2012 ▸). Similarly, in the brain, the direction of the neuronal axons is used to infer structural connectivity (Johansen-Berg & Rushworth, 2009 ▸), and aberration in structural and functional networks is associated with neuropathologies (Sundgren *et al.*, 2004 ▸; Xie & He, 2011 ▸; Bakshi *et al.*, 2008 ▸; Grefkes & Fink, 2014 ▸; Cao *et al.*, 2015 ▸). A variety of techniques to investigate 3D tissue organization have been developed over the past few years (Georgiadis, Müller *et al.*, 2016 ▸). However, most of them are restricted either to the analysis of tissue sections such as polarized light imaging (Axer *et al.*, 2011 ▸; Bromage *et al.*, 2003 ▸) and 3D scanning small-angle X-ray scattering (Georgiadis *et al.*, 2015 ▸), or to very small sample volumes such as in volume light and electron microscopy (Helmstaedter *et al.*, 2008 ▸; Briggman & Bock, 2012 ▸; Reznikov *et al.*, 2013 ▸).

Methods that can be used to retrieve the 3D orientation of micro- and nanostructure in tissue sections can be extended to volumetric analyses if multiple contiguous sections are scanned and then stacked together, as was shown by Georgiadis *et al.* (2015 ▸) and Georgiadis, Müller *et al.* (2016 ▸), in which the 3D orientation of collagen fibers was represented with a 3D vector for each voxel in the entire bone trabecula. However, not all sample studies are amenable to thin sectioning, either because it limits further studies or because of potential artifacts introduced by physically cutting the sample.

Large sample volumes can be studied with SAXS- (small-angle X-ray scattering) or XRD- (X-ray diffraction) CT (computed tomography) (Birkbak *et al.*, 2015 ▸; Poulsen, 2012 ▸; Jensen *et al.*, 2011 ▸), although the orientation-dependent scattering is not fully accounted for. Thus, these methods can only provide a statistical description of the nanostructure orientation, and not a 3D orientation map, as in Skjønsfjell *et al.* (2016 ▸), Mürer *et al.* (2018 ▸). An alternative method for examining large samples is diffusion-weighted magnetic resonance imaging (DWI), which assesses the anisotropic diffusivity of tissue water as a proxy for tissue structural organization (Alexander *et al.*, 2007 ▸), *e.g.* of the orientation of white matter fibers in the brain, or of collagenous fibers in muscles, tendons or ligaments. However, DWI is limited to the study of the diffusion of molecules with high overall mobility, *e.g.* water molecules in tissue. Also, since diffusion is only a proxy for assessing microstructure, structural interpretation may be ambiguous (Jones *et al.*, 2013 ▸), resulting in a constant need for validation (Hubbard & Parker, 2009 ▸; Dell’Acqua & Catani, 2012 ▸).

Alternatively, information on structural organization can be derived from X-ray small-angle scattering tensor tomography (SASTT). Recently, three tensor reconstruction techniques based on directional data from X-ray scattering were introduced (Malecki *et al.*, 2014 ▸; Liebi *et al.*, 2015 ▸; Schaff *et al.*, 2015 ▸). These techniques extend X-ray tomography, yielding not only a scalar value, but also a set of parameters characterizing 3D local anisotropic nanostructure in every voxel. Analogous to CT, data are acquired by rotating the sample around a tomography axis. Yet, in addition, the rotation axis is tilted at various angles resulting in a distributed coverage of sample orientations relative to the incident X-ray beam (Liebi *et al.*, 2018 ▸).

Tensor reconstruction approaches are based on 2D projections of the sample from different orientations, where for each point in the projection 2D information about the structure orientation has been collected. These approaches work for imaging in different orientation-sensitive contrast modalities such as grating interferometry (Malecki *et al.*, 2014 ▸), light (Gandjbakhche *et al.*, 1994 ▸; Girasole *et al.*, 1997 ▸), neutron (Hongladarom *et al.*, 1996 ▸) or X-ray scattering (Gourrier *et al.*, 2010 ▸; Georgiadis *et al.*, 2015 ▸; Liebi *et al.*, 2015 ▸; Bünger *et al.*, 2010 ▸).

In the approach of Malecki *et al.*, a grating interferometry setup is used. In this case, grating interferometry is sensitive to the micro- or nanostructure oriented perpendicular to the grating orientations; thus, projections need to be acquired with different relative orientations between the gratings and the sample around the beam direction (Malecki *et al.*, 2014 ▸). In contrast, for small-angle scattering the information on 2D orientation is measured directly on the 2D scattering pattern. The SASTT reconstruction technique presented by Liebi *et al.* modeled anisotropy in every voxel by a superposition of spherical harmonics (Liebi *et al.*, 2015 ▸, 2018 ▸). Model parameters were optimized by minimizing the error between the intensities predicted by the model and experimental values for all projections measured. The procedure yields two direction angles, which represent the nanostructure principal orientation, and the coefficients of the spherical harmonics that describe the shape of the reciprocal-space map for every voxel. Schaff *et al.* (2015 ▸) define a virtual axis of rotation *a posteriori* for each direction of the 3D reciprocal-space map that is to be reconstructed and then find the projection angles and 2D scattering orientation that would align to this ‘virtual axis’; as there will be no perfect match to the required orientations, an error threshold for the sample orientation angles is introduced. Finally, they invoke the principle of rotation invariance (Feldkamp *et al.*, 2009 ▸) to reconstruct these components of the 3D reciprocal-space map using standard tomography algorithms. This process is repeated for each virtual axis in 3D space. After reconstruction, the 3D reciprocal-space map in each voxel is fitted to an ellipsoid tensor model to parametrize the local nanostructure anisotropy and orientation in 3D. Both tensor tomography reconstruction techniques suffer from limitations: first, they are computationally intense, resulting in a time- and resource-consuming reconstruction process. In addition, the approach based on spherical harmonics (Liebi *et al.*, 2015 ▸) requires an educated guess to improve convergence speed and avoid local minima, to which gradient descent methods are prone. The method based on virtual rotation axes (Schaff *et al.*, 2015 ▸) requires a dense angular sampling of projection orientations for identifying the rotationally invariant component of the nanostructure in each voxel, potentially leading to an unnecessary trade-off between measurement time and accuracy. Hence, a reconstruction algorithm with significantly reduced computational time and without the above limitations is highly attractive.

We present a method for tomographic reconstruction of the 3D nanostructure organization, iterative reconstruction tensor tomography (IRTT), which is fast, efficient and robust. For IRTT reconstruction we use a symmetric second-rank tensor to model tissue anisotropy in every voxel, similar to the model widely used in diffusion tensor imaging (DTI) (Mori, 2014 ▸), and previously used for X-rays (Malecki *et al.*, 2014 ▸; Seidel *et al.*, 2012 ▸). An iterative method similar to the algebraic reconstruction technique (ART) (Gordon *et al.*, 1970 ▸) is introduced. The method is fast and efficient due to the use of the symmetric second-rank tensor, which only needs six parameters to describe local 3D structure anisotropy, and the introduction of a linearized reconstruction strategy. In order to extend ART from scalars to a six-parameter tensor model, the method of iterative error backpropagation (Rumelhart *et al.*, 1986 ▸), inspired by the training process of artificial neural networks (Hecht-Nielsen, 1989 ▸, 1992 ▸), is applied in the reconstruction. In this article, the IRTT method is presented in detail. The feasibility and accuracy of IRTT reconstruction are demonstrated with three samples exhibiting different properties: a knot made from carbon fibers, a human bone trabecula and a fixed mouse brain. The results are both qualitatively and quantitatively compared with those obtained with the SASTT method (Liebi *et al.*, 2015 ▸, 2018 ▸).

## 2. Methods   

### 2.1. Experimental setup for data acquisition   

**Figure 1:**
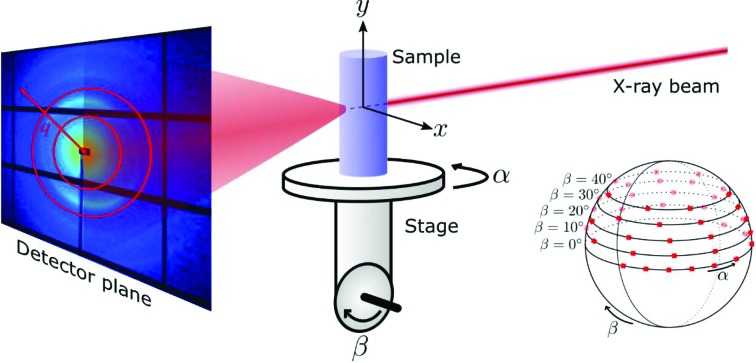
IRTT experimental setup, similar to that in Liebi *et al.* (2015 ▸), Schaff *et al.* (2015 ▸). The sample is raster-scanned 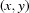 by a pencil-beam for different rotation and tilt angles α and β. Best practice is to distribute the angles α and β homogeneously on the hemisphere of sample orientations (Liebi *et al.*, 2018 ▸), as shown on the inset sphere. The red arrow on the detector indicates a *q* vector in reciprocal space, and the two red circles enclose an example *q* range.

X-ray scattering experiments were performed in the coherent small-angle X-ray scattering (cSAXS) beamline of the Swiss Light Source, Paul Scherrer Institute (PSI), Villigen, Switzerland, which is fitted for fast position-resolved scanning SAXS and SASTT experiments. In the experimental setup, shown in Fig. 1 ▸, the sample is mounted on a computer-controlled stage that can translate in two directions (*x*, *y*) in the plane of the detector and rotate around two axes corresponding to rotation angle α and tilt angle β. The sample is scanned through the beam and a Pilatus photon-counting detector (Henrich *et al.*, 2009 ▸), positioned downstream, records the SAXS patterns. The detector position and angular coverage determine the investigated *q* range in reciprocal space; illustrated with a red arrow in the detector plane of Fig. 1 ▸ is a particular *q* vector, while a possible *q* range to investigate is enclosed between two red circles. The *q* range defines the characteristic range of *d* spacing in physical space according to the relation 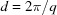.

Each scattering pattern corresponds to the cumulative scattering from all voxels along the X-ray beam path through the sample, as shown in Fig. 1 ▸. To capture a full 2D projection, the sample needs to be raster-scanned in two directions 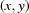 across the beam. At the same time, the sample transmission is recorded by a photodiode that also acts as a beamstop to prevent damage to the detector by the directly transmitted beam. The translation step size is typically matched to the beam size and desired resolution. After scanning all the points in the defined field of view (FOV), the sample is rotated to the next projection angles 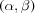.

To record 2D projections from all possible directions, and probe the 3D volume of the sample, the stage is controlled in the following way: for a tilt angle β of the tomographic axis, the sample is rotated around the axis and the angle α takes values 0° 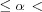 360°. At β = 0°, only rotations for α in the range of 0 to 180° are required, similar to parallel-beam CT. The range of rotation from 0° to 360° for the non-zero tilt angles makes negative tilt angles redundant (Liebi *et al.*, 2018 ▸). The total number of projections and their distribution, *i.e.* the total number of rotation and tilt angle sets 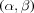, depends on the sample diameter, structural complexity, desired spatial resolution and available scan time. Best practice is to distribute all projection angles in a uniformly distributed angular grid about the hemisphere of directions (Liebi *et al.*, 2018 ▸), as shown in the inset sphere in Fig. 1 ▸.

### 2.2. Samples   

The reconstruction algorithm was applied to three samples: carbon fibers tied into a knot, a bone trabecula extracted from a human vertebra and a fixed mouse brain. Details concerning sample preparation and experiments can be found in Appendices *A* [App appa] and *B* [App appb].

**Figure 2:**
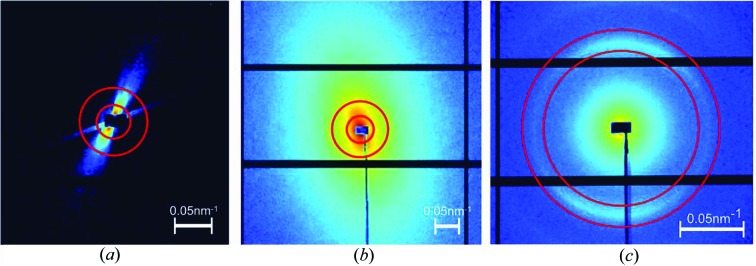
Scattering patterns from the three samples. Red circles represent the analyzed *q* ranges. (*a*) The carbon fiber knot, (*b*) trabecular bone, (*c*) fixed mouse brain.

Typical scattering patterns from the three samples are shown in Fig. 2 ▸; the red circles indicate the analyzed *q* range. Numerical values for the *q* ranges used for analysis can be found in Table 1 (see Appendix *B* [App appb]).

### 2.3. Symmetric intensity reconstruction and segment analysis   

**Figure 3:**
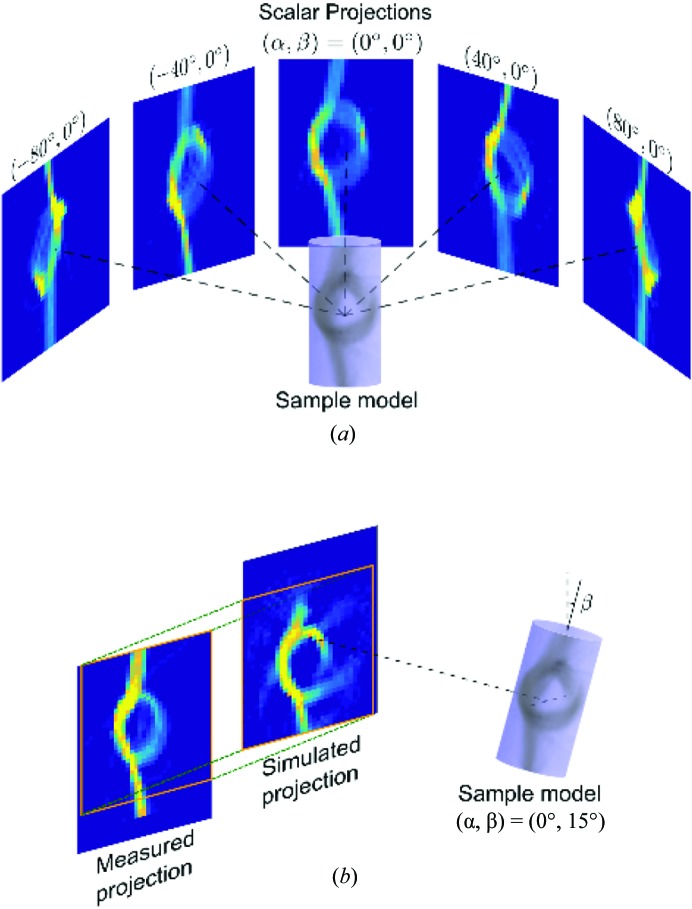
Scalar projections of the signal intensity and corresponding reconstruction, from which a 3D support for the reconstruction can be obtained. (*a*) Projections for different angles α and β = 0°. (*b*) 2D registration of the measured projection to the one derived from the 3D model for a specific set of angles 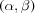.

The first step of IRTT is the reconstruction of the 3D geometry of the sample using a scalar quantity. Either the projection of the absorption coefficient or the azimuthally averaged scattering intensity can be used for this purpose. The simplest approach for reconstructing either of the two quantities is to use filtered back-projection, given enough projections at β = 0°, and then use this initial 3D sample model to align all projections, as shown in Fig. 3 ▸: since the sample is in practice not perfectly aligned in the rotation center of both rotation and tilt axes, each measured projection with 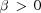 is registered to the 3D scalar model similar to the procedure of Liebi *et al.* (2018 ▸). This is achieved by computing 2D projections from the 3D scalar model for every 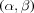 as a template for the registration of experimental projections for the same orientation using a 2D subpixel image registration algorithm (Guizar-Sicairos *et al.*, 2008 ▸), as exemplified in Fig. 3 ▸(*b*).

**Figure 4:**
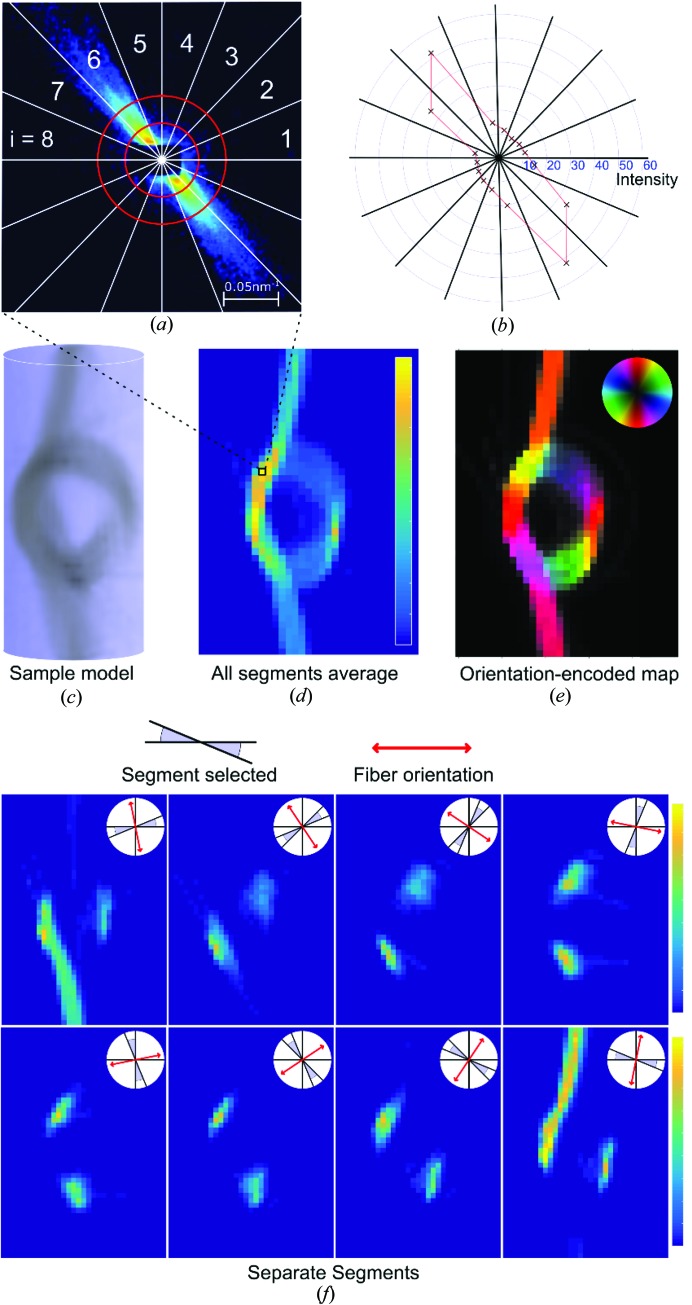
Anisotropy analysis of scattering patterns. (*a*) The scattering pattern is divided into 16 azimuthal segments and the desired *q* range is chosen for investigation. (*b*) The intensity of each segment is integrated and subsequently reduced to eight segments, exploiting the center symmetry of the scattering pattern. (*c*) 3D scalar model of the carbon fiber knot. (*d*) Intensity map displaying the average intensity from all eight segments for the (0°, 0°) projection. (*e*) Analysis of anisotropic intensity across segments for all the points of one projection yields a 2D orientation-encoded map, in which each pixel is assigned a color corresponding to the main fiber orientation according to the inset color wheel. (*f*) 2D scattering intensity maps of the eight segments for the (0°, 0°) projection. The segment and corresponding fiber orientation are depicted as insets. The fiber orientation, shown in red, is assumed to be perpendicular to the bisector of the angular segment.

In-plane nanostructure orientation is derived from the azimuthal intensity variation in each scattering pattern. The anisotropy of each pattern is analyzed by dividing the detector frame into angular segments (Bunk *et al.*, 2009 ▸). We have used 16 segments to discretize the azimuthal intensity, but in principle any number sufficient to capture the in-plane anisotropy can be chosen for this purpose. We then select a *q* range that corresponds to characteristic dimensions of the structure of interest, as shown in Fig. 4 ▸(*a*), and the scattering intensity of each of the 16 segments at this specific *q* range is visually depicted in Fig. 4 ▸(*b*). Furthermore, the number of segments can be averaged and reduced to eight by exploiting the center symmetry of SAXS patterns, and the resulting intensity distribution per segment is shown in Fig. 4 ▸(*f*). These eight intensity values for every point of each projection constitute the input for the IRTT algorithm, as described in the following sections.

The orientation-encoded maps, for example as shown in Fig. 4 ▸(*e*), serve in this article as 2D visualizations of the data. To calculate these maps, we use a method similar to that presented by Bunk *et al.* (2009 ▸). Specifically, for each diffraction pattern, the azimuthal segment intensity values are analyzed using a discrete Fourier transform to extract the 2D orientation and the isotropic and anisotropic intensity components (Bunk *et al.*, 2009 ▸). When performed for all the points in one projection, this yields a 2D fiber orientation map that is shown in Fig. 4 ▸(*e*), where the orientation, degree of orientation and intensities are visualized using a hue-saturation-value representation (Fratzl *et al.*, 1997 ▸). In the latter each measured point of the sample is represented by a pixel which is assigned a color corresponding to the main fiber orientation according to the inset color wheel.

### 2.4. Tensor model for each voxel   

In SAXS experiments, anisotropic scattering is related to anisotropy of the sample structure (Klug & Alexander, 1954 ▸). For example, for the carbon knot sample, the anisotropic intensity of the scattering signal mainly originates from edge scattering at the interface between the carbon fibers and air, so that the main scattering is oriented perpendicular to the fiber bundle, as shown in Fig. 4 ▸(*f*).

**Figure 5:**
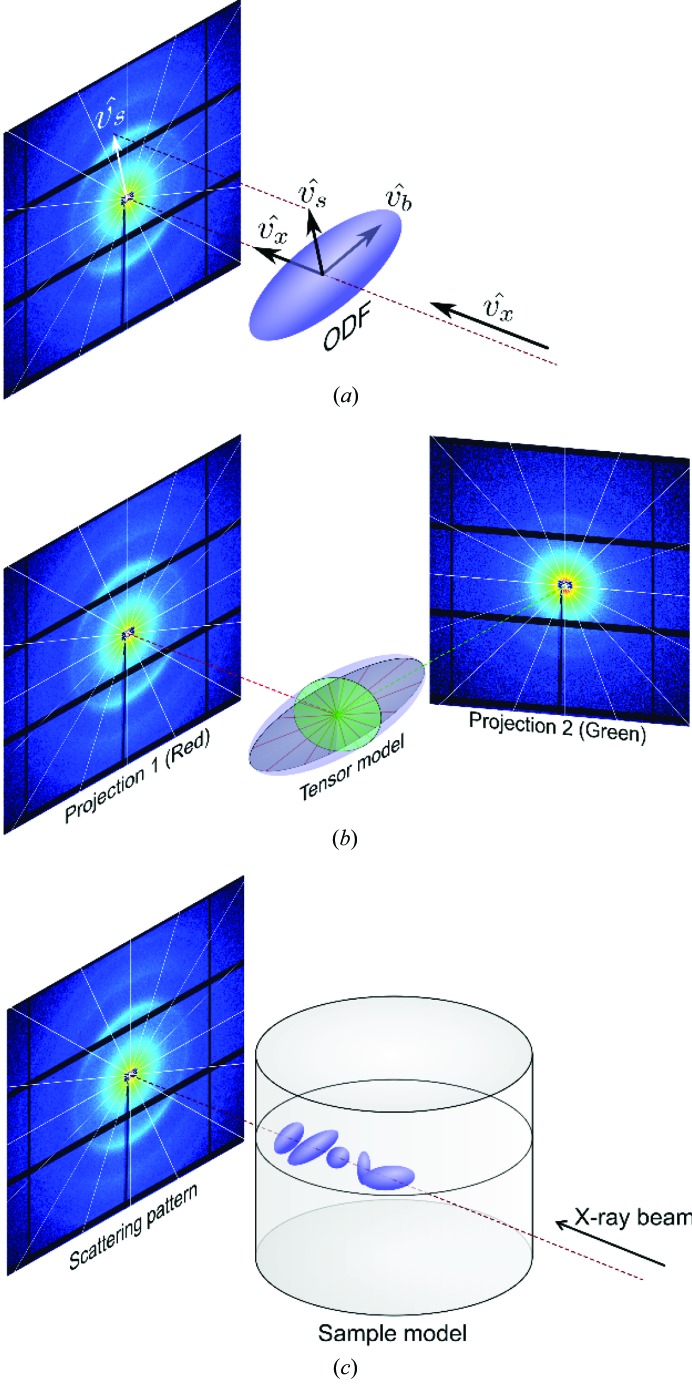
Scattering based on a tensor model for sample nanostructure organization (scattering patterns shown are from the brain sample, further discussed in Section 3.3). (*a*) Relationship between the X-ray propagation direction 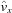, nanostructure orientation distribution function (ODF) and measurement orientations 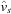. (*b*) The direction 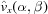 of the incident X-ray beam and the segment vectors 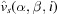 on the detector plane perpendicular to the beam direction are defined by the angles α and β. The measured scattering pattern depends on the projection angles, and the measurement is described by an elliptical section of the ODF in a plane parallel to the detector plane. For a cross section for which the long ellipsoid axis is larger than its short axis, such as the red elliptical section in the ODF, there will be a highly anisotropic intensity distribution along the azimuthal direction in the detector for the corresponding *q* radius. On the other hand, for a section with both axes of similar length (green elliptical section) the azimuthal intensity distribution on the scattering pattern will be almost isotropic. (*c*) As there is one ODF tensor per voxel, the measured scattering pattern constitutes the sum of contributions of the individual ODF tensors along the path of the X-ray beam through the sample.

The relationship between the direction of the beam, the sample and the detector segment is illustrated in Fig. 5 ▸(*a*), where 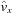 is the direction unit vector of the incoming X-ray beam, 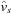 the direction unit vector of a detector segment *i*, which is defined by its bis­ector, and 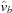 the unit vector of the corresponding direction of the nanostructure orientation distribution function (ODF), which generates scattering in that segment. Here, we assume that the maximum intensity observed in the angular segment *i* is generated by sample nanostructure with its principal orientation 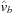 perpendicular to the direction, as is the case for the samples and *q* ranges used in this study.

Without loss of generality we can assume that the coordinate frame is fixed to the sample. Then, for each projection 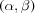 the beam and detector are rotated and tilted around it, as exemplified in Fig. 5 ▸(*b*), *i.e.* the IRTT method as presented employs object-centric coordinates. Given the above-mentioned perpendicularity between 2D scattering and nanostructure for the measured samples, one can calculate the structure orientation as^1^

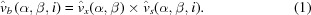
IRTT assumes that the local nanostructure ODF can be described by an ellipsoid, or in mathematical terms, by a second-rank 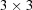 tensor 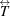. The measured intensity 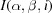, along 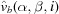, can be expressed as 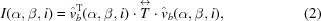
where 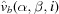 is given by equation (1) for any value *i*.

Given enough projections well distributed around the hemisphere of sample orientations, as shown in the inset sphere of Fig. 1 ▸, the tensor 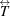 that best fits the data can be found.

Because the scattering patterns are center symmetric, the tensor 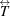 is symmetric (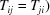, comprising six independent components only, 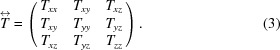
If the three components of 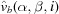 are written as 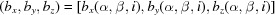, then equation (2) can be expanded: 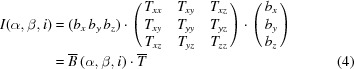
where 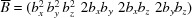 incorporates all the terms related to the experimental geometry, and 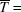 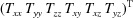 incorporates all the tensor components related to the sample nanostructure.^2^

Treating the individual segments separately allows us to define the intensity vector 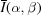, 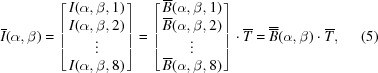
where 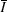 comprises the intensity information for all segments, and 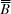 all vectors related to the measurement geometry in equation (1).

### 2.5. Tensor tomography reconstruction   

For SASTT (Liebi *et al.*, 2015 ▸; Schaff *et al.*, 2015 ▸), the scattering pattern from each pixel of a 2D projection is the result of the beam passing through multiple voxels, *i.e.* probing a complex combination of nanostructure at different orientations. Hence, each scattering pattern is composed of the sum of contributions from all the voxel-associated tensors along the beam path: 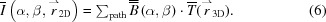
Here, 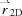 is the position vector of the point in the projection plane, and 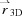 is the position vector of each voxel in the sample. Since 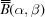 only depends on the projection angles, it can be moved outside of the sum, and we can replace the sum along the beam path with the sum over the whole volume using a Dirac delta function, 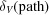, which selects all voxels in the beam path, 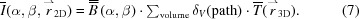
In practice, contributions from all voxels along the beam path are summed according to the proximity of their center to the beam using trilinear interpolation.

Equation (7) constitutes the basis of the reconstruction algorithm. The array 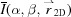 represents experimental data for different segments, 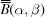 is the predefined matrix that relates segment intensities to the tensor in each voxel depending solely on the projection angle set 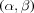, and 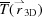 is the tensor model to be reconstructed.

In order to find the appropriate tensor 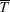 for each voxel, we need to minimize the difference between the simulated 3D model projections 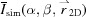, calculated using equation (7), and the measured projections, 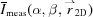. Different optimization algorithms, such as gradient descent, can be used to perform such an error minimization. Here, in order to enhance the convergence speed, we applied an approach similar to the ART, a class of iterative reconstruction methods commonly used in tomographic reconstructions (Kak & Slaney, 1988 ▸). In ART, the model is reconstructed iteratively by back-projecting the difference between the measured projections and those obtained from the current 3D model. Yet, in tensor tomography, a back-projection cannot be performed without considering the 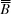 term which encodes the effects of directional scattering. In IRTT, the solution for this problem was inspired by error backpropagation (Hecht-Nielsen, 1992 ▸, 1989 ▸), a method that originated in the field of artificial neural networks (Zell *et al.*, 1993 ▸).

**Figure 6:**
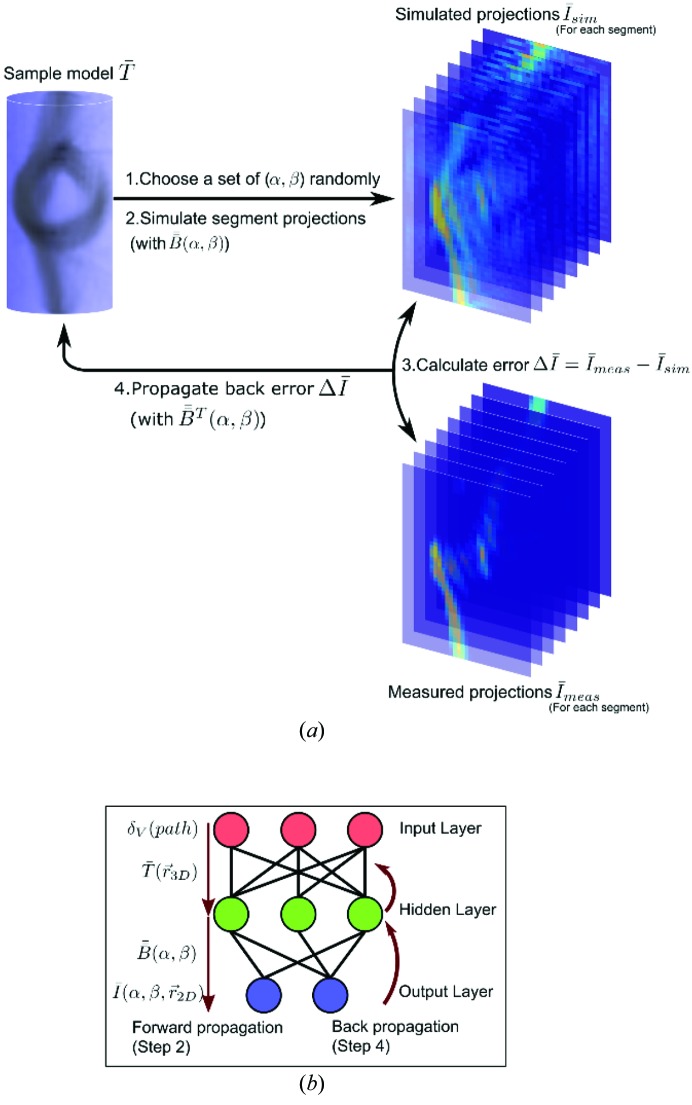
(*a*) Flow chart illustration of the iterative reconstruction method. (*b*) Illustration of a double-layer artificial neural network, as applied in IRTT.

The method is schematically described in Fig. 6 ▸(*a*). If we treat equation (8) as a double-layer neural network, Fig. 6 ▸(*b*), the Dirac delta function, *i.e.* the input layer, selects the tensors 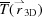 of voxels along the path, giving a computational intermediate result, shown as the hidden layer. This result propagates to the output layer, comprising the scattering intensities, through 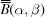, which selects the corresponding terms in the tensor ODF to be reflected in the scattering intensity of these projection angles 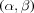. The objective is to optimize 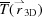, *i.e.* the connection weights between input and hidden layer. This is achieved by taking the error in the output layer of scattering intensity, comparing it with the measured intensity, and propagating their difference backwards through the second layer of connections 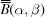 by taking its transpose 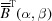. The backpropagated error is used to correct the first layer of connections 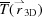, shown in Fig. 6 ▸(*b*) as the backward direction. Tensor tomographic reconstruction is achieved based on this error backpropagation through an iterative ART scheme.

Before starting the iterative procedure, an initial tensor model is assigned to all the voxels of the structure. For this initial tensor model, we have tried to use all-zeros, random numbers and isotropic tensors scaled by the symmetric intensity information retrieved as described in Section 2.3. With numerous trials we found that all these different initial models converge consistently to the same solution, *i.e.* with a coefficient of variation, defined as the ratio of standard deviation to mean value, of less than 0.04 for non-air voxels. In view of the method’s robustness to the initial model, we have chosen the simplest, all-zero model 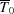 as the default starting point for all reconstructions.

In every iteration a random set of angles 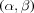 is chosen from the experimental set of angles. A simulated projection 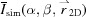 is then computed from the 3D sample model, compared with the measured projection, 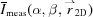, and their difference 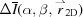 = 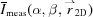 − 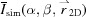 calculated.

In order to make a correction on the reconstructed model 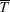 based on the error 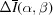, the update function, 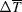, is obtained by solving for 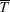 in equation (7), namely: 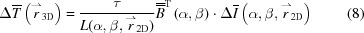
where τ is the so-called ‘correction ratio’ that denotes the percentage of the error 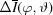 that is propagated back to the model (Hecht-Nielsen, 1989 ▸). While the exact value of τ influences the final result, a value around 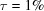 proved to be very robust and was used for all samples in this study. 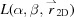 is the number of sample voxels in the beam path in equation (7).

Using equation (8), all tensors in the sample volume can be corrected along the angles (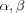). The deviation of the simulated values from the measured ones is minimized in an iterative way, each time with a new, randomly selected single projection from the set of (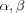).

In order to evaluate the goodness of reconstruction of reconstructed models, an error parameter ∊ was defined as the root-mean-square of the difference between modeled and measured intensity values of all experimental projections 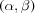: 
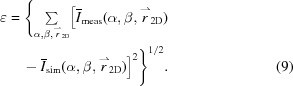
All the codes used for the reconstruction algorithm described in this article are available at https://doi.org/10.5281/zenodo.1480589.

## 3. Results and discussion   

### 3.1. Carbon fiber knot   

The carbon fiber knot has a highly anisotropic scattering, as shown in Fig. 2 ▸(*a*), which mainly arises from surface scattering of individual fibers, *i.e.* it originates from their ‘macroscale’ arrangement. The degree of scattering anisotropy is significantly higher for such a sample than is typically encountered for structured biological samples such as mineralized collagen fibrils in bone or myelinated axons in the nervous system. However, as the fiber orientation in the carbon fiber knot is known at a macroscopic scale, it serves as a test sample for method validation.

**Figure 7:**
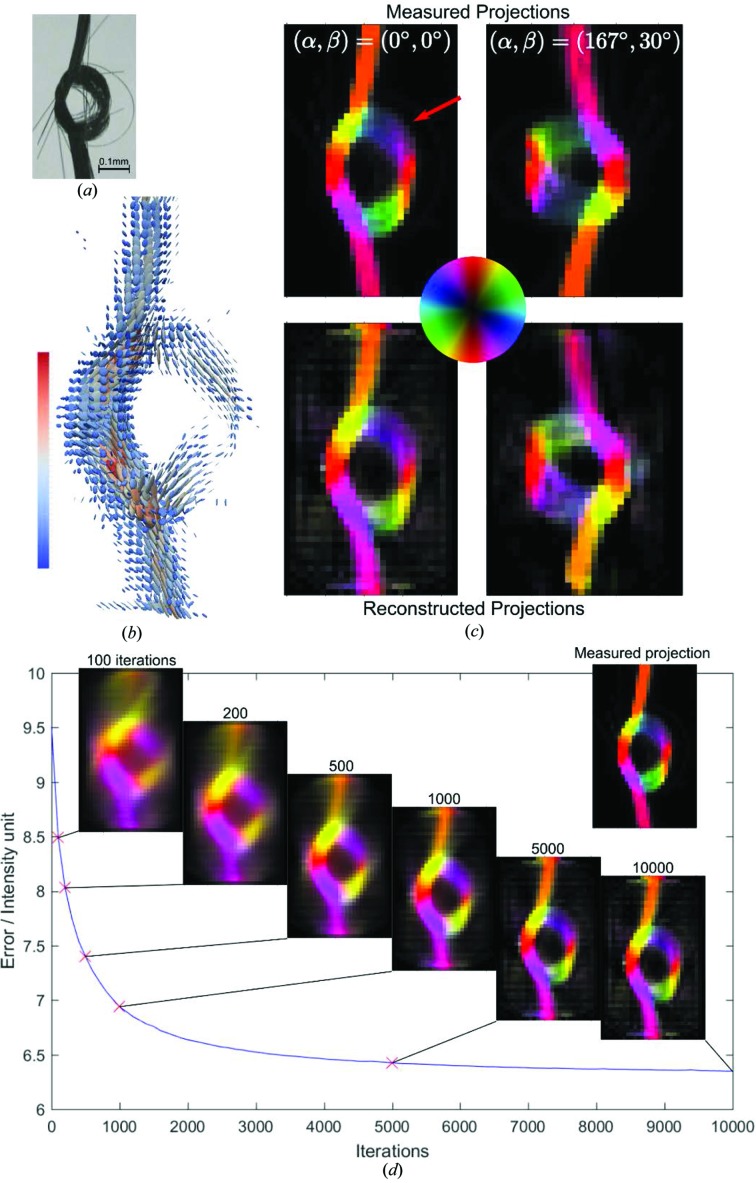
IRTT reconstruction of the carbon fiber knot. (*a*) Optical image of the fiber knot, where regions of loose fiber packing are visible. (*b*) Results of the tensor reconstruction visualized as 3D ellipsoids. The color bar indicates the tensor magnitude in linear scale and arbitrary units. (*c*) Experimental and IRTT-derived orientation-encoded maps are shown in the upper and lower panels, respectively, for two sample orientations. The red arrow points to a region of loosened fibers. Orientation is color-coded by the color wheel. (*d*) Error evolution versus iteration number, and orientation-encoded reconstructed projections for selected iteration numbers. The measured projection is also shown as an inset.

The optical photograph of the fiber knot, Fig. 7 ▸(*a*), reveals the macroscopic carbon fiber arrangement. Experimentally determined orientation-encoded images are shown in Fig. 7 ▸(*c*) (upper panels) for two sample orientations 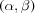, and a video containing images for all the orientations can be found in the supporting information. The area of reduced intensity in the projection 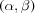 = (0°, 0°), indicated by a red arrow in Fig. 7 ▸(*c*), is due to less dense, loosened fibers. Fig. 7 ▸(*b*) shows the second-rank tensor ODF obtained with IRTT for every voxel, visualized by an ellipsoid. Each tensor represents the local nanostructure ODF; the eigenvectors of the tensor matrix define the orientation of the axes of the ODF ellipsoid, and therefore carry information about the main orientation directions of the nanostructure. The associated eigenvalues, 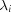, define the length of the major axes of the ellipsoid, such that the total scattering is proportional to 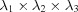 and the degree of orientation is encoded in how different these values are, *i.e.* the eccentricity of the ellipsoid. Most tensors in the carbon knot reconstruction are very anisotropic, with the first eigenvalue an order of magnitude larger than the other two, owing to the very dominant direction of the fibers and the resulting strong scattering streaks in the scattering patterns. To evaluate the quality of the fit, we compared the simulated projections, *i.e.* projections computed using the reconstructed model, shown in the lower panels of Fig. 7 ▸(*c*), with the experimentally measured results for the same values of (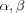), shown in the upper panels of Fig. 7 ▸(*c*). At a first glance, there is a high level of correspondence regarding both the degree of anisotropy and the fiber orientation. A more careful observation reveals subtle differences. For instance, the simulated projections show a higher background signal compared with the experimental data. We attribute this discrepancy to the high degree of orientation of the sample. The model based on ellipsoid tensors can only account for variations with cosine dependence with respect to the azimuthal angle. This approximation fails for nano­structures with a very high degree of anisotropy such as aligned carbon fibers, and a more complex model such as higher orders of spherical harmonics may be needed to capture more precisely this level of anisotropy. Another possible explanation for the background signal might be the ‘missing wedge’ artifact, analogous to that observed in transmission electron microscope tomography (Kováčik *et al.*, 2014 ▸; Tam & Perez-Mendez, 1981 ▸; Carazo, 1992 ▸), due to the fact that not all rotation angles α are accessible for measurement because of physical constraints of the experimental setup, *i.e.* the rectangular frame supporting the knot.

Fig. 7 ▸(*d*) shows the evolution of the error given in equation (9) versus the iteration number, and the corresponding reconstructed signal from all segments at selected iteration numbers for the 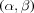 = (0°, 0°) projection. The measured projection is shown for reference. The error quickly drops during the first 1000 iterations, and the knot shape clearly appears. Thereafter, the rate of improvement slows down until, at around 10 000 iterations, the algorithm essentially reaches convergence. Typically, good reconstruction quality was achieved at around 10 000 iterations for most samples. We note that one iteration is defined as every time a single 2D projection is used to update the tensors via equation (9). The residual error can be attributed to (i) the noise in the experimental data, (ii) the inability of the tensor model to fully capture the scattering anisotropy, (iii) other sources of error introduced in the data analysis procedure, *e.g.* slight errors in registration or inaccuracies in scanning positions.

Overall, the IRTT algorithm provided a fast and accurate reconstruction that agrees with our prior knowledge of the carbon fiber knot sample. The reconstruction of 38 686 voxels took 67 s for 10 000 iterations on a single thread of an Intel Xeon Gold 6140 CPU @ 2.30 GHz processor.

The data set and reconstruction results for the carbon fiber knot sample are available at https://doi.org/10.5281/zenodo.1480589.

### 3.2. Human trabecular bone   

**Figure 8:**
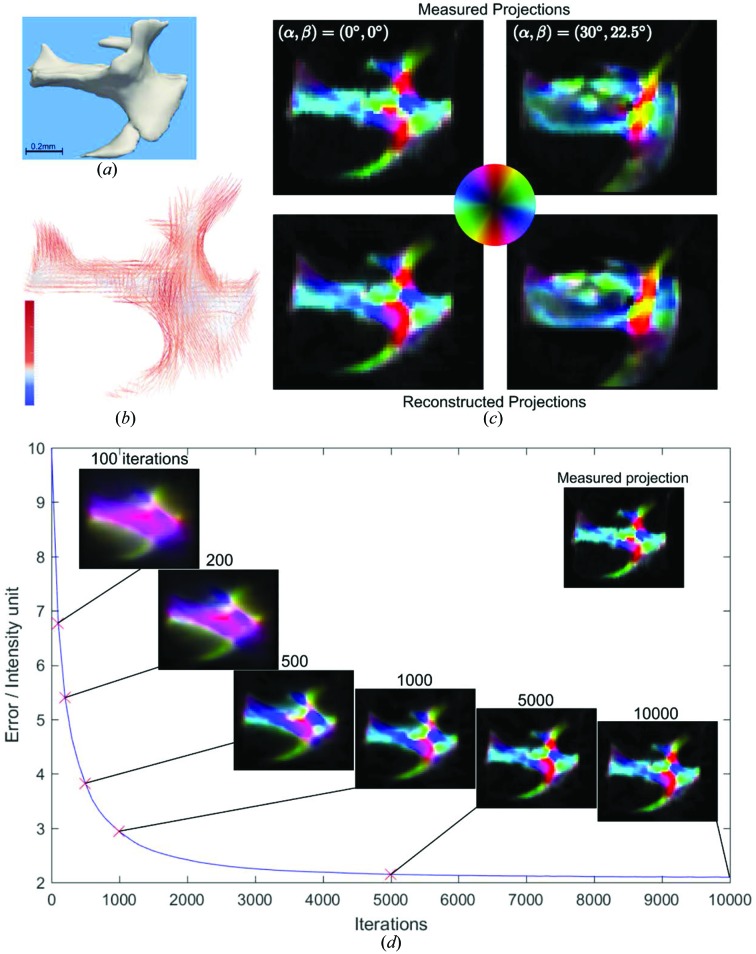
IRTT reconstruction result of trabecular bone specimen from human vertebral body. (*a*) Volume-rendered model of 3D sample geometry. (*b*) 3D representation of the principal eigenvector of the ODF. The color bar indicates the corresponding eigenvalue in linear scale and arbitrary units. (*c*) Experimental and IRTT-derived orientation-encoded maps are shown in the upper and lower panels, respectively, for two sample orientations. Orientation is color-coded by the color wheel. (*d*) Error evolution with iterations and corresponding reconstructed orientation-encoded maps for (α, β) = (0°, 0°). Note the improved edge definition as the number of iterations increases. For reference, the corresponding experimental orientation-encoded map is shown as an inset.

Mineralized collagen fibers in bone have a complex organization, the 3D structure of which has become the topic of recent studies (Reznikov *et al.*, 2014 ▸; Georgiadis, Guizar-Sicairos *et al.*, 2016 ▸; Liebi *et al.*, 2015 ▸) due to its relevance to micromechanical properties and bone pathologies (Martin & Ishida, 1989 ▸; Granke *et al.*, 2013 ▸; Giannini *et al.*, 2012 ▸; Gourrier *et al.*, 2010 ▸). Given the high electron-density differences between the mineral crystals and the surrounding medium, pronounced anisotropy can be observed in the scattering signal from bones, *cf*. Fig. 2 ▸(*b*). The IRTT reconstruction results for the trabecular bone specimen of a human vertebra, the same sample shown in Liebi *et al.* (2018 ▸), are displayed in Fig. 8 ▸. A volume-rendered view of the trabecular structure is shown in Fig. 8 ▸(*a*). The experimental and reconstructed orientation-encoded intensity maps of two projections at different sample orientations are shown in Fig. 8 ▸(*c*), and a video of all experimental projections can be found in the supporting information. The measured projections reveal a complex nanostructure organization with many domains of tens of micrometres in size, as expected from previous studies of human trabecular bone (Georgiadis, Guizar-Sicairos *et al.*, 2016 ▸; Georgiadis *et al.*, 2015 ▸). Fig. 8 ▸(*b*) shows a line rendering, in which for each voxel we show a line in the direction of the first eigenvector corresponding to the largest eigenvalue, and hence of the main orientation of the nanostructure, with the color indicating the length of that eigenvalue. We choose this representation, instead of the ellipsoids of Fig. 7 ▸(*b*), for visual clarity, due to the larger number of reconstructed tensors in the sample. The 3D fiber direction map reveals domains with strong and weak main fiber orientation. Fiber direction apparently follows the trabecular microstructure, especially regions exhibiting high curvature as has been shown for trabecular bone (Georgiadis, Guizar-Sicairos *et al.*, 2016 ▸). The goodness of fit can be visually assessed by comparing the simulated and measured projections, shown in Fig. 8 ▸(*c*) in the lower and upper panels, respectively. Similar to the carbon knot results, these show a high degree of similarity, although small differences can be observed. These differences likely originate from the limitations of the second-rank tensor model to fully describe an arbitrary 3D degree of orientation. The evolution of the residual error as a function of the number of iterations, Fig. 8 ▸(*d*), shows a similar trend as for the carbon fiber knot case; in both cases the larger structures and their directionality become apparent within the first few hundred iterations. In the next few thousand iterations features are optimized, and smaller spatial domains are revealed. The time required for the reconstruction of 228 150 voxels from 240 projections with 10 000 iterations was 5.6 min on a single thread of an Intel Xeon Gold 6140 CPU @ 2.30 GHz processor.

The data set and reconstruction results for the human trabecular bone sample are available at https://doi.org/10.5281/zenodo.1480589.

### 3.3. Reconstructing the orientation of myelinated fibers in mouse brain   

**Figure 9:**
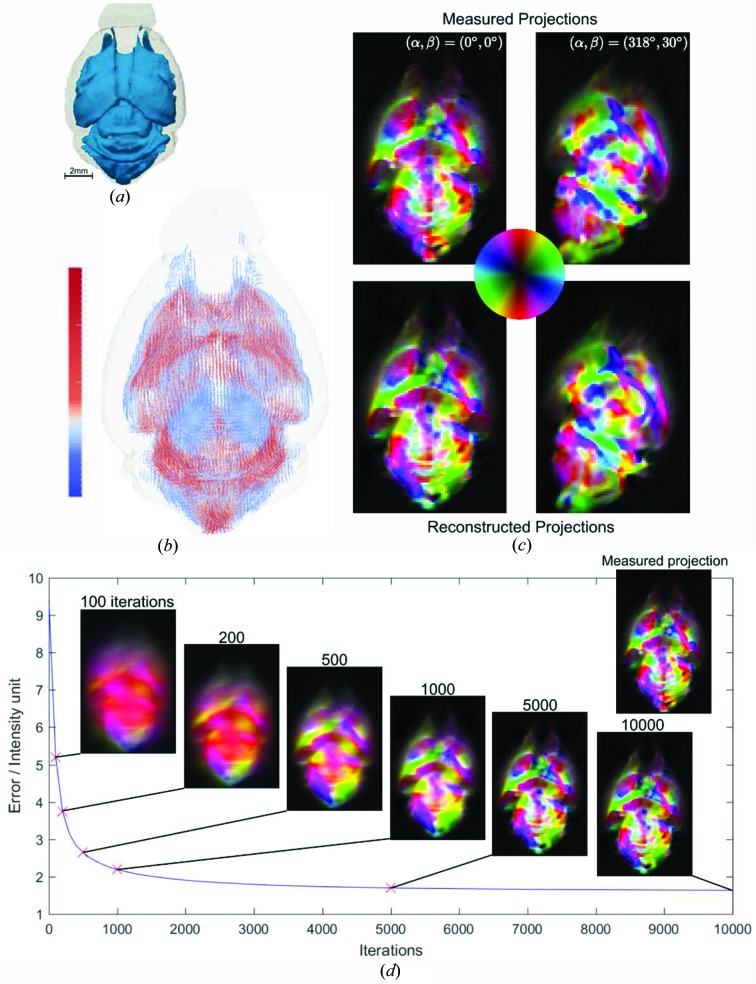
IRTT reconstruction of a fixed mouse brain. (*a*) Volume-rendered model displaying the distribution of the structure of interest, *i.e.* myelin, within the brain specimen. (*b*) 3D representation of the ODF principal eigenvector. The color bar indicates the corresponding eigenvalue in linear scale and arbitrary units. (*c*) Experimental and IRTT-derived orientation-encoded maps are shown in the upper and lower panels, respectively, for two sample orientations. (*d*) Error evolution as a function of iteration number and corresponding reconstructed projection for (α, β) = (0°, 0°). Note that edge definition increases with the number of iterations. For reference, the corresponding experimentally measured orientation-encoded projection is shown as an inset.

Structural connectivity in the brain is based on myelinated fibers connecting distinct brain areas (Azevedo *et al.*, 2009 ▸). The study of these connections has recently become the subject of extensive ‘connectomics’ research (Sporns *et al.*, 2005 ▸). Because of its relative simplicity, *i.e.* the lack of cortical gyration, the rodent brain has become an attractive subject for studying structural and functional connectivity (Grandjean *et al.*, 2017 ▸). Myelin exhibits a characteristic scattering peak in SAXS, as shown in Fig. 2 ▸(*c*), which corresponds to a physical period or *d* spacing of ∼17 nm, and which allows both its spatial distribution and orientation to be mapped (Jensen *et al.*, 2011 ▸; Georgiadis, Gao, Zingariello *et al.*, 2017 ▸; Georgiadis, Gao, Liebi *et al.*, 2017 ▸). Mapping the myelin distribution in the mouse brain reveals the major myelinated brain areas, *i.e.* the brain white matter, as shown in a surface-rendered view in Fig. 9 ▸(*a*). Two projections from the experimental data reveal a highly complex white matter fiber orientation [see the upper panel of Fig. 9 ▸(*c*)]. For comparison, the simulated projections from the IRTT reconstruction are depicted for the same azimuthal and tilt angles in the lower panels of Fig. 9 ▸(*c*). The directions of the eigenvectors corresponding to the largest eigenvalues of the ODF tensors from each voxel are shown in Fig. 9 ▸(*b*) and indicate the principal orientation of the nano­structure. These directions are consistent with the known neural fiber directions in the mouse brain, *e.g.* with fibers running along the olfactory tracts, the optic tracts and the corpus callosum (Georgiadis, Gao, Zingariello *et al.*, 2017 ▸; Georgiadis, Gao, Liebi *et al.*, 2017 ▸). The goodness of fit can be evaluated by visually comparing simulated and measured projections as shown in Fig. 9 ▸(*c*). It should be noted that IRTT is by default unable to truthfully reconstruct nanostructure ODF in voxels comprising fibers of different directionality, such as fiber crossings, for which more complex models such as a set of spherical harmonics are required (Liebi *et al.*, 2015 ▸, 2018 ▸). This is analogous to diffusion MRI-based approaches, where limitations of tensor-based approaches become obvious for certain brain regions, and more complex model functions have been suggested to capture the heterogeneity of the anisotropy of water diffusion (Tuch, 2004 ▸; Fernandez-Miranda, 2013 ▸; Farquharson *et al.*, 2013 ▸; Novikov *et al.*, 2018 ▸).

Similar to samples shown in the previous sections, the principal structural features of the brain and their orientations have been reconstructed using less than 1000 iterations, see Fig. 9 ▸(*d*). The reconstruction of 514 500 voxels with 267 projections converges after approximately 10 000 iterations, which were achieved in 15.7 min on a single thread of an Intel Xeon Gold 6140 CPU @ 2.30 GHz processor.

### 3.4. Comparison of IRTT with SASTT   

IRTT and SASTT (Liebi *et al.*, 2015 ▸) differ with regard to the model used for representing nanostructure organization in each voxel and the reconstruction algorithm. In SASTT, the spatial anisotropy within a voxel can be obtained from the reciprocal-space map, which is modeled using a set of spherical harmonics and a vector representing a principal orientation of the nanostructure. In contrast, the model used by IRTT consists of a second-rank tensor with six independent parameters, which constitutes a simple way of representing the distribution of nanostructure orientations within one voxel, including secondary anisotropy perpendicular to the dominant direction. Yet, the model is limited in its representation of complex nano­structure arrangements such as multiple fiber directions, as occur for example with crossing or kissing fibers within one voxel. In contrast, spherical harmonics provide a complete basis that can be used to represent any anisotropy distribution, provided terms of sufficiently high order are included in the set. It is noteworthy that the SASTT method demonstration in Liebi *et al.* (2015 ▸) employs only even-order spherical harmonics, similar to advanced diffusion MRI methods (Frank, 2001 ▸; Tuch *et al.*, 2002 ▸; Tuch, 2004 ▸) with 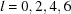; however, in Liebi *et al.* (2015 ▸) only 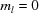 were used, thereby assuming cylindrical symmetry. Accordingly, in its demonstrations thus far SASTT utilizes six independent parameters, equal to the tensor model: the four coefficients for the spherical harmonics plus two angles defining the principal orientation of the nanostructure in 3D space. The assumption of cylindrical symmetry in Liebi *et al.* (2015 ▸) limits the possibility to model a secondary anisotropy in the plane perpendicular to the dominant direction; this in principle could be alleviated by optimization of higher *m* orders. However, this would increase the number of unknowns in the reconstruction and remains to be tested. On the other hand, including spherical harmonics to the sixth order allows for a more complex representation of anisotropy along the principal direction, which can capture for instance sharper peaks, as shown in Fig. 6 in Liebi *et al.* (2018 ▸), or complex scattering features as shown in Fig. 7 in Liebi *et al.* (2018 ▸) for the *q* value corresponding to the collagen peak.

The second important difference between IRTT and SASTT is the reconstruction algorithm. SASTT uses a gradient descent method (Cauchy, 1847 ▸). Six independent parameters are updated in every iteration by a gradient descent algorithm that minimizes an error function, which quantifies the difference between the current model prediction and the experimental data. However, gradient descent’s linear search can be slow, in particular if the problem is ill-conditioned (Greenstadt, 1967 ▸; Akaike, 1998 ▸), and is also susceptible to converging to local minima. In contrast, the IRTT algorithm is based on the concept of error backpropagation (Rumelhart *et al.*, 1986 ▸) which can quickly identify the structural components that need to be corrected. It also uses an iterative procedure: in every iteration step the model is adjusted considering one randomly chosen experimental projection only. This allows reconstruction of the whole 3D model by a simple process and faster convergence is achieved. In SASTT, the initial model needs to be defined with the symmetric intensity and an additional regularization step is used to improve convergence and to avoid stagnation in local minima (Liebi *et al.*, 2015 ▸, 2018 ▸). These steps are not necessary for IRTT, which shows a robust convergence towards a unique solution.

The fact that the reconstruction is completely linearized, along with the robustness of the tensor model, renders the algorithm robust and significantly faster than previous methods (Liebi *et al.*, 2015 ▸, 2018 ▸).

**Figure 10:**
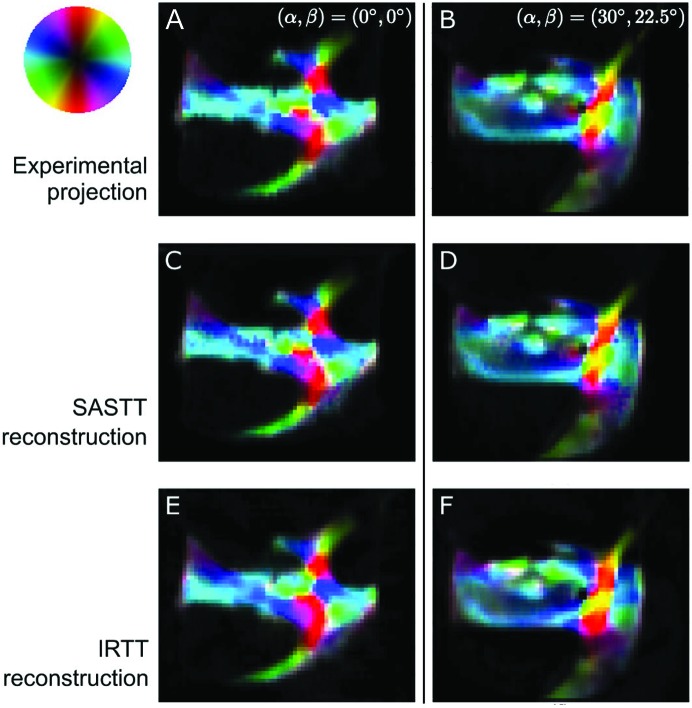
Comparison of IRTT and SASTT in reconstructing anisotropy of the trabecular bone specimen, for two representative projection angles. (*a*)–(*b*) Experimental projections showing orientation-encoded maps of the bone trabecular sample. (*c*)–(*d*) SASTT-reconstructed projection displaying intensities for the same orientations. (*e*)–(*f*) IRTT-reconstructed projections. 2D nanostructure orientation can be interpreted by the color wheel.

A comparison of the reconstruction results of IRTT and SASTT is shown in Fig. 10 ▸, where the experimental and reconstructed projections for the trabecular bone specimen for two angle sets (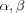) are displayed. Overall, both methods reproduce the main features of the projection anisotropy reasonably well: the physical dimensions, the directionality and the degree of anisotropy of the individual sample domains are captured by both SASTT and IRTT. However, it appears that spherical harmonics shows better performance in reproducing fine details. This visual observation is also supported by the quantitative overall error (∊) as given in equation (9). The final error was 2.27 for SASTT, 2% lower than the error of 2.32 given for IRTT.

**Figure 11:**
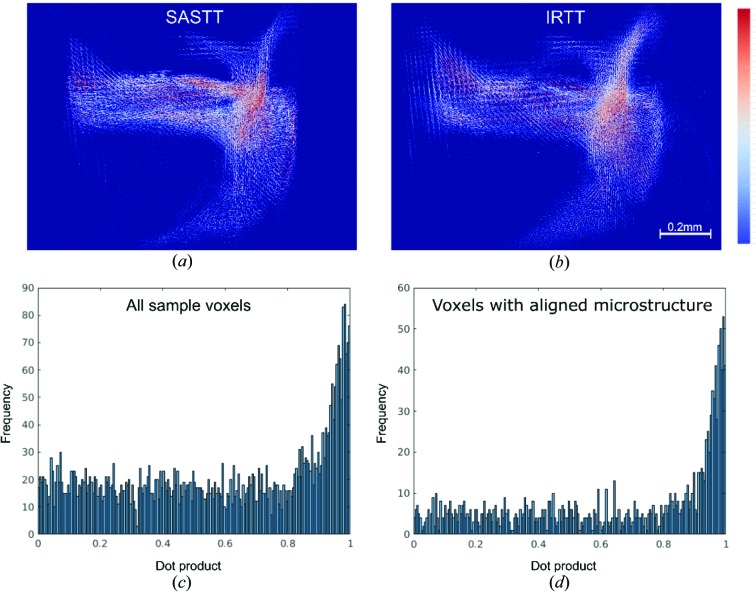
Quantitative comparison of IRTT and SASTT for the trabecular bone specimen. (*a*)–(*b*) 3D visualization of the main anisotropy direction: first eigenvector 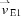 reconstructed by IRTT (*a*), and 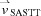 points along the principal direction of anisotropy from SASTT (*b*). (*c*) Histogram of the dot product of unit vectors 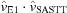 across the whole sample. (*d*) Histogram of the dot product for voxels displaying clear directionality 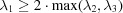.

For more detailed comparison between the direction results from IRTT and SASTT, the dot product of the first eigenvector 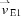 results reconstructed by IRTT and the vector 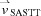 pointing along the principal direction of anisotropy derived from SASTT was computed for the trabecular bone specimen. For reference, a dot product equal to one signifies a perfect correspondence of the directions reconstructed by the two methods. Visual inspection of the orientation-encoded maps shown in Figs. 11 ▸(*a*) and 11 ▸(*b*) reveals good qualitative agreement. The dot product histogram across all sample voxels, excluding voxels in air, shown in Fig. 11 ▸(*c*), is skewed towards 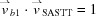, showing a high level of agreement between results of the tissue anisotropy analysis from the two methods. The agreement becomes stronger when considering only voxels with pronounced nanostructure anisotropy, defined here as the ratio between the largest and second largest eigenvalue being 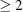 (Fig. 11 ▸ *d*). Computing the dot product of this subset of voxels results in a distribution highly skewed towards 1.

### 3.5. Comparison of reconstruction time   

The trabecular bone specimen was used to benchmark and compare the new IRTT method against SASTT non-linear optimization reconstruction. Reconstructions were carried out in one node of the Ra cluster at the Paul Scherrer Institut. The node has 12 dual-core Intel Xeon E5-2690v3 processors (2.60 GHz) and 256 GB of RAM. The reconstruction time for SASTT was 128 min, including all the steps outlined in Liebi *et al.* (2015 ▸, 2018 ▸), *i.e.* optimization of the symmetric intensity, of the initial values of the two angles defining the principal orientation, and jointly the coefficients of the spherical harmonics and the aforementioned angles using regularization, each with 50 iteration steps. It should be noted that the procedure currently used for determining the regularization constant using an L curve (Liebi *et al.*, 2018 ▸) is time consuming, 38 min for this case, which in total adds up to 166 min for the SASTT reconstruction of the trabecular bone sample. In contrast, IRTT reconstruction with 10 000 iterations took 4.5 min, which is a remarkable speed-up of more than one order of magnitude. Values in this range have also been confirmed for other test samples.

### 3.6. Limitations and outlook   

The novel IRTT reconstruction method is an efficient tool for reliably capturing anisotropic tissue structure. Nevertheless, there are some limitations inherent to the model and algorithms used. Firstly, the second-rank tensor model, which can be represented by an ellipsoid, is not ideally suited for capturing complex anisotropy distributions within a single voxel, *e.g.* fibers crossing or kissing or a very high level of anisotropy such as the parallel aligned carbon fibers. In such cases, more complex models based on multiple orders of spherical harmonics can be better suited. Higher nano­structure complexity may require the use of spherical harmonics of azimuthal orders 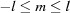; in that case we would give up the cylindrical symmetry approximation while retaining the center symmetry assumption, similar to DWI approaches (Frank, 2001 ▸; Tuch *et al.*, 2002 ▸; Tuch, 2004 ▸).

Concerning the number of iterations needed for convergence, in the current study we have selected a number (10 000) which yielded good results for all samples tested. Further studies are needed to develop strategies for finding an optimal iteration number, which will probably be sample dependent. Also, no regularization strategies have been used so far, which however might be required when going to higher iteration numbers, since we expect semi-convergence phenomena known to iterative reconstruction techniques (Tommy *et al.*, 2014 ▸). Future studies will investigate this.

Concerning reconstruction time, since computations for error backpropagation are calculated independently along each beam trajectory, the speed of both methods could be significantly enhanced by using graphical processing units (GPU), a step planned for the future.

The method of iterative error backpropagation, which here is used for the rank-2 tensor representation, could in principle be applied to reconstruction methods using a spherical harmonics model, such as SASTT. This would provide an alternative to the gradient descent approach and potentially enhance its computation and convergence speed; work in this direction is currently ongoing.

Although IRTT has been used in this article for modeling and reconstructing the nanostructure ODF directly, it could also be used to model and reconstruct the reciprocal-space sample scattering in each voxel, similar to SASTT, as explained in the footnote in Section 2.4.

IRTT has been proven to be fast and robust in recovering the principal orientation of nanostructures, a very time-consuming step in SASTT (Liebi *et al.*, 2018 ▸), which in part relies on *a priori* knowledge or assumptions on the sample structure (Liebi *et al.*, 2018 ▸). Based on the methodological differences between the two methods, it becomes very attractive to combine them, *e.g.* using IRTT as a first-line analysis followed by SASTT to refine the results. For this reason, IRTT may become particularly useful not only as a fast and robust stand-alone reconstruction method, but also in (i) providing an initial guess for SASTT or other reconstruction algorithms, thereby reducing the overall computational load, and (ii) enabling online reconstructions for feedback on data quality and completeness during an experiment.

## 4. Conclusion   

IRTT is introduced here as a novel, fast and robust method for tomographic reconstruction of the anisotropic nano­structure organization inside materials and tissues. IRTT uses experimental 2D anisotropy information in projections measured for multiple sample orientations (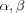). The reconstruction is based on a tensor model for describing the ODF within each voxel. Model parameters are optimized by iterative backpropagation of the difference between experimental and reconstructed data for all voxels for a randomly chosen projection at each iteration step. IRTT has been shown to be more than an order of magnitude faster compared with previously described reconstruction algorithms (Liebi *et al.*, 2015 ▸, 2018 ▸). This is due to (i) the use of a simpler physical model characterizing the structural anisotropy, *i.e.* a second-rank tensor versus spherical harmonics used in SASTT, and (ii) an optimization algorithm that employs linearization and error backpropagation to update the model based on a single projection for each iteration cycle. IRTT might be used as a robust tensor tomography reconstruction method, for examining anisotropic nanostructure in materials and tissues. Additionally, its speed makes it suitable for use as a quick first-line reconstruction method for identifying the main nanostructure orientation within each voxel, which can then be used as a starting point for a more refined or general reconstruction such as SASTT. This would significantly reduce the overall reconstruction time by eliminating the multiple steps needed for SASTT and by significantly reducing the number of iterations required to refine the solution.

Feasibility studies with different samples such as a highly oriented artificial material, as well as hard and soft tissue specimens, revealed that IRTT yields accurate and robust reconstructions in an efficient manner. A complete tensor tomography pipeline based on the IRTT algorithm described in this article might constitute an attractive tool for studying microstructural anisotropy in material sciences and in biomedical research.

## Supplementary Material

Video of carbon knot orientation-encoded projections. DOI: 10.1107/S2053273318017394/vk5029sup1.avi

Video of trabecular bone orientation-encoded projections. DOI: 10.1107/S2053273318017394/vk5029sup2.avi

## Figures and Tables

**Table 1:** Experimental information for the SAXS scans of the three analyzed samples

Sample	*A* (carbon knot)	*B* (human trabecula)	*C* (mouse brain)
Beam energy (keV)	12.4	12.4	16.3
Beam size (µm)	30 × 20	25 × 25	150 × 75
Motor step (= voxel) size (µm)	40	25	150
Detector distance (m)	7	7	2
No. of projections	249	240	267
Scanning (FOV) matrix	29 × 46	55 × 65	70 × 105
Scanning (FOV) size (mm)	1.16 × 1.84	1.38 × 1.63	10.5 × 15.75
Analyzed *q* range (nm^−1^)	0.021–0.049	0.038–0.076	0.64–0.84
Exposure time/point (ms)	30	30	120
Total exposure time (h)	2.8	7.2	65
Measurement time (h)	8.5	20.3	82

## Footnotes

1 It should be noted that, although IRTT, as presented in this article, is used to model and reconstruct the nanostructure ODF, it can also be used to reconstruct the reciprocal-space sample scattering from each voxel, similar to SASTT (Liebi *et al.*, 2015 ▸, 2018 ▸), by simply using  instead of equation (1), with the rest of the steps remaining the same.

2 This is analogous to the equations describing the orientation-dependent signal intensity in diffusion MRI (Mori, 2014 ▸), with , where  comprises the experimental parameters including the directional angles  under which the diffusion process is interrogated, while the diffusion tensor  corresponds to .

## Appendix A Sample preparation

Sample *A* consists of a bundle of carbon fibers (CF-Roving HT 24 K, from Suter-Kunststoffe AG) tied into a knot. Both ends of the fibers were fixed onto a rectangular metal frame to expose the knot in the middle.

Sample *B* is a trabecula from a T12 human vertebra from a 73-year-old man, also described in Liebi *et al.* (2018 ▸). It was extracted and all soft tissue removed before embedding into polymethylmethacrylate (PMMA). The vertebra specimen was obtained from the Department of Anatomy, Histology and Embryology at the Innsbruck Medical University, Innsbruck, Austria, with the written consent of the donor according to Austrian law.

Sample *C* is a fixed whole brain specimen from a 5-month-old female C57BL/6 mouse. All procedures were carried out according to the Swiss Federal Law for Animal Protection and approved by the Veterinary Authorities of the Kanton of Zurich. After the mouse was anesthetized, it was trans­cranially perfused with phosphate-buffered saline (PBS) and 4% paraformaldehyde (PFA) in PBS, and the brain was extracted, PFA-fixed overnight at 4°C (277 K), and stored in PBS at 4°C. For the SAXS scan, the fixed brain was embedded in PBS-based 1% agarose gel inside a Kapton tube (1 cm in diameter, 140 µm wall thickness, Goodfellow Cambridge Ltd, Huntingdon, UK).

## Appendix B Experiments

Measurements were performed at the cSAXS beamline (X12SA) of the Swiss Light Source, Paul Scherrer Institute, Switzerland. The X-ray beam was monochromated by a fixed-exit double-crystal Si(111) monochromator, and focused horizontally, *i.e.* sagittally, by bending the second monochromator crystal and vertically, *i.e.* meridionally, by bending a Rh-coated mirror. To minimize air scattering and X-ray absorbance a 7 m (for samples *A* and *B*) or 2 m (for sample *C*) evacuated flight tube was positioned between the sample and detector. The X-ray scattering was measured with a Pilatus 2M detector (Henrich *et al.*, 2009 ▸) and the transmitted beam measured simultaneously by a diode mounted on a beamstop placed inside the flight tube.

Experimental details for the three samples can be found in Table 1 ▸. For sample *A*, a few rotation angles around α = ±90° had to be excluded due to the supporting metal frame blocking the view onto the sample. For retrieving the myelin-specific signal in sample *C*, the background scattering was subtracted by fitting an inverse power-law function . Then, the peak height from Gaussian peak fitting at *q* = 0.74 ± 0.04 nm^−1^, the *q* range of the strongest myelin peak, was used in order to retrieve the scattering of myelin, a procedure similar to that described by Agrawal *et al.* (2009 ▸) and Jensen *et al.* (2011 ▸). Fig. 2 ▸ depicts one representative scattering pattern from each sample, together with its analyzed *q* range, also indicated in Table 1 ▸.
